# Supplementary material for: Integrative Analysis Identifies Candidate Tumor Microenvironment and Intracellular Signaling Pathways that Define Tumor Heterogeneity in NF1
Source: Genes (Basel). 2020 Feb 21;11(2):226. doi: 10.3390/genes11020226 (PMC7073563; doi:10.3390/genes11020226)
Supplement: Supplementary file 1 [file genes-11-00226-s001.zip › Genes_NF1_SupplementaryMaterials/Updated_Figure S2.pdf]

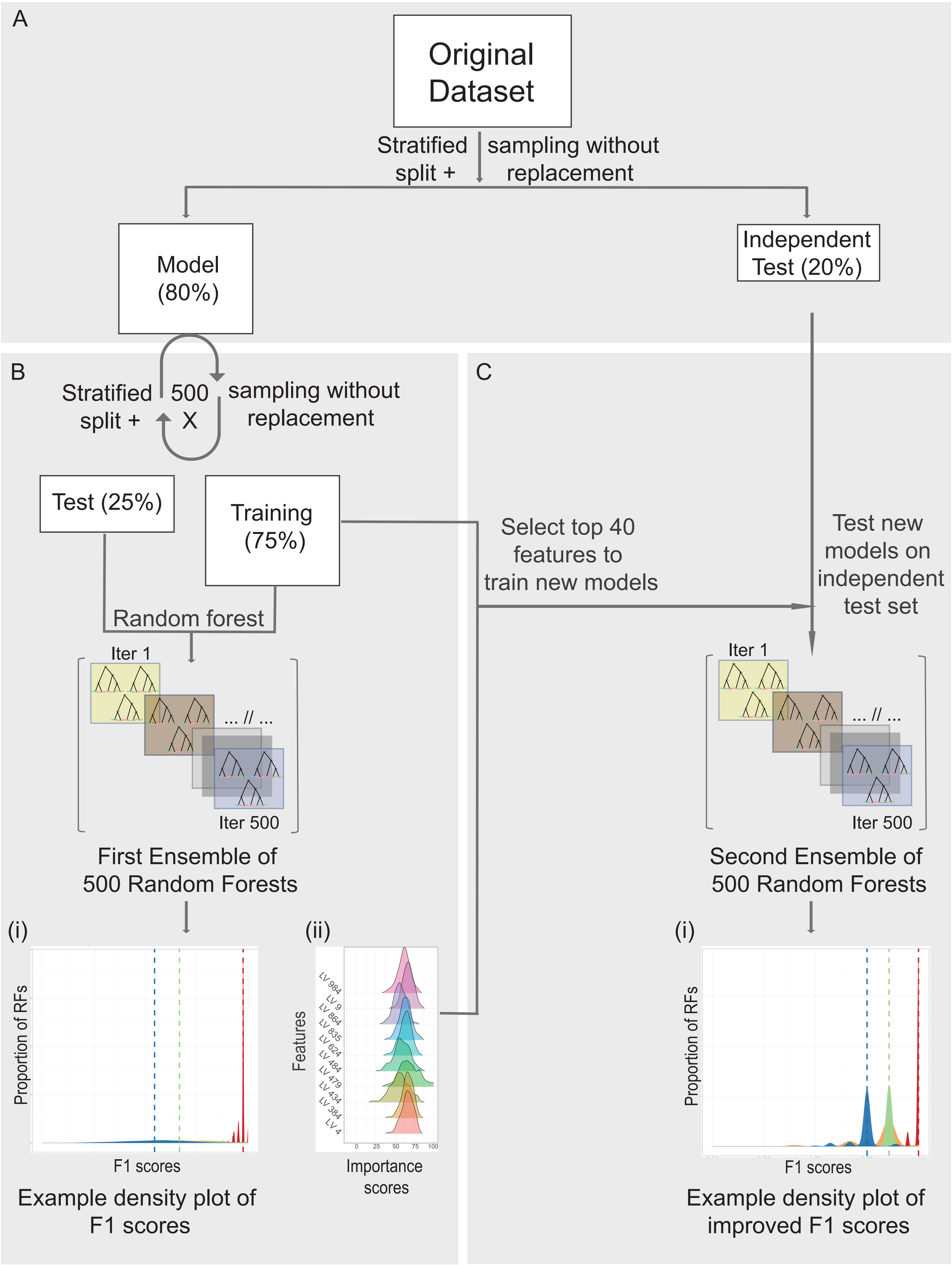
A
Original Dataset
Stratified split +
sampling without replacement
Model (80%)
Independent Test (20%)
B
Stratified split +
500 X
sampling without replacement
Test (25%)
Training (75%)
Random forest
Iter 1
... // ...
Iter 500
First Ensemble of 500 Random Forests
(i)
Proportion of RFs
F1 scores
Example density plot of F1 scores
(ii)
Features
LV 984
LV 9
LV 864
LV 835
LV 624
LV 484
LV 479
LV 434
LV 384
LV 4
0
25
50
75
100
Importance scores
C
Select top 40 features to train new models
Test new models on independent test set
Iter 1
... // ...
Iter 500
Second Ensemble of 500 Random Forests
(i)
Proportion of RFs
F1 scores
Example density plot of improved F1 scores
